# Diagnostic value of MRI for detecting recurrent soft-tissue sarcoma in a long-term analysis at a multidisciplinary sarcoma center

**DOI:** 10.1186/s12885-021-08113-y

**Published:** 2021-04-13

**Authors:** Sam Sedaghat, Maya Sedaghat, Jens Meschede, Olav Jansen, Marcus Both

**Affiliations:** 1 Department for Radiology and Neuroradiology, University Hospital Schleswig-Holstein Campus Kiel, Arnold-Heller-Str. 3, 24105 Kiel, Germany; 2 Institute of Diagnostic and Interventional Radiology and Nuclear Medicine, University Hospital Bergmannsheil, Bürkle de la Camp-Platz 1, 44789 Bochum, Germany; 3 Department for Radiology and Neuroradiology, Klinikum Dortmund, Beurhausstraße 40, 44137 Dortmund, Germany

**Keywords:** Diagnostic accuracy, MRI, Soft-tissue sarcoma, Long-term analysis, Sarcoma center

## Abstract

**Background:**

Soft-tissue sarcomas (STS) are rare tumors of the soft tissue. Recent diagnostic studies on STS mainly dealt with only few cases of STS and did not investigate the post-therapeutic performance of MRI in a routine clinical setting. Therefore, we assessed the long-term diagnostic accuracy of MRI for detecting recurrent STS at a multidisciplinary sarcoma center.

**Methods:**

In all, 1055 postoperative follow-up MRIs of 204 patients were included in the study. MRI follow-up scans were systematically reviewed for diagnostic values (true-positive/−negative and false-positive/−negative results) in detecting recurrences. Pathological reports and follow-up MRIs were set as baseline references.

**Results:**

The median age of the patients was 55.3 ± 18.2 years. Of the patients, 34.8% presented with recurrences. Here, 65 follow-up scans were true positive, 23 false positive, 6 false negative, and 961 true negative. The overall sensitivity and specificity of MRI for detecting recurrences were 92 and 98%, respectively, with an accuracy of 97%. For intramuscular lesions and after surgery alone the sensitivity was higher (95 and 97%, respectively) than for subcutaneous lesions and surgery with additional radiation therapy (83 and 86%, respectively), at similarly high specificities (96–98%). The 6 false-negative results were found in streaky (*n* = 2) and small ovoid/nodular (*n* = 4) recurring lesions. The false-positive lesions imitated streaky (*n* = 14), ovoid/nodular (*n* = 8), and polycyclic/multilobulated recurring tumors (*n* = 1). All false-positive results were found in patients in whom the primary tumors were polycyclic/multilobulated in appearance.

**Conclusion:**

MRI shows a high diagnostic accuracy for detecting recurrent STS, with a high sensitivity and specificity. The diagnostic accuracy decreases in subcutaneous lesions and after surgery with radiation therapy, compared to intramuscular lesions and surgery alone. Radiologists should pay particular attention to streaky and small ovoid/nodular recurring lesions and patients with polycyclic/multilobulated primary tumors.

## Background

Soft-tissue sarcomas (STS) constitute a rare and heterogeneous group of tumors, accounting for only 1% of adult malignancies [1]. Due to the rarity of these malignancies, there is often only little experience in dealing with STS and in postoperative surveillance outside of specialty centers [2]. More than 50 different subtypes have been described, and the extremities are the most common sites of STS [1, 3]. Surgery is the most common treatment option for STS, with additional radiotherapy in selected cases [4]. In the literature, different strategies for postoperative surveillance of STS patients have been reported. Nevertheless, a unified strategy is still lacking [1, 5–7]. The most commonly used imaging modalities for the post-therapeutic follow up of STS patients are ultrasound, computed tomography (CT), and magnetic resonance imaging (MRI). While CT is usually used for the screening of distant metastasis, ultrasound and MRI are used for detecting local recurrences [8, 9]. Studies on the diagnostic accuracy of MRI in detecting STS already exist. Nevertheless, recent studies on this topic mostly included only few cases of STS and did not examine the performance of post-therapeutic MRI in a routine clinical setting [10–12]. Therefore, we analyzed the diagnostic value of MRI for detecting recurrent STS in the long-term, postoperative follow-up at a multidisciplinary sarcoma center. Furthermore, we analyzed whether the localization of STS in the soft tissue or the type of therapy has an impact on the diagnostic value of MRI.

## Methods

### Patients

**Fig. 1:**
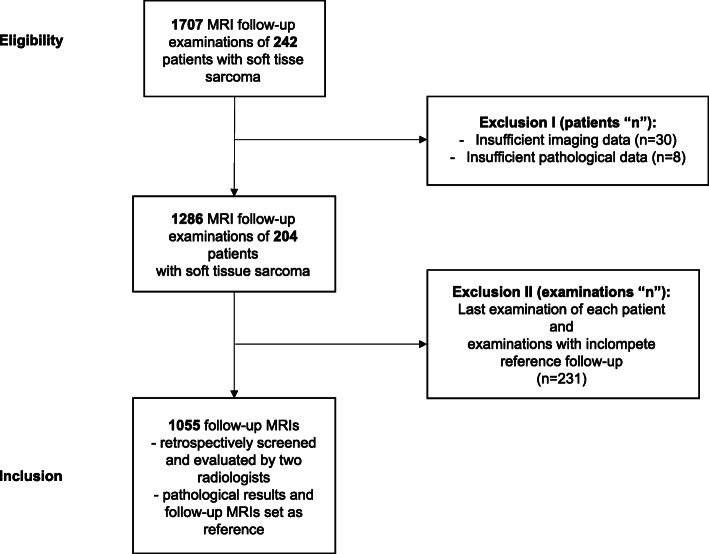
Overview of the study design with inclusion and exclusion criteria

A total of 1707 postoperative follow-up MRI scans were performed in 242 patients with histologically proven STS between 2008 and 2020. Thirty-eight patients were excluded due to insufficient imaging and pathological data. Ultimately, 204 patients with a total of 1286 postoperative follow-up MRI scans were included in our study and presented complete data on imaging. Examinations in which predictive values could not be determined and the last examination of each patient were excluded (*n* = 231; Fig. 1). Either core needle or open biopsy was performed in all lesions suspected of being recurrences (*n* = 88). In these patients, the pathological reports were set as reference and the radiological findings were correlated to the pathological reports. All other MRIs were reviewed during the subsequent MRI follow-up examinations, showing whether lesions had been overlooked in the previous MRIs. These subsequent MRI follow-ups took place after 3 to 6 months. The follow-up MRIs were reviewed by two dedicated musculoskeletal radiologists with a minimum of 5 years of experience in sarcoma diagnostics, with findings reached by consensus. The reports were divided into two groups: presence of recurrence and absence of recurrence. From these findings we extracted true-positive/−negative and false-positive/−negative MRI findings in detecting recurrent STS. The false-negative results were derived retrospectively by reviewing the subsequent MRI follow-ups. Latest recurrences were clearly delimited after two such follow-ups.

The pre-established standard schedule for MRI examination of all patients was four times in the first year (every 3 months), twice in the second post-therapeutic year (every 6 months), and once a year for a minimum of three consecutive years thereafter. The first MRI examination routinely took place 3 months after primary tumor resection. Nevertheless, 173 patients of the 204 patients included in total strictly adhered to the MRI follow-up examinations that were provided. Thirty-one patients partially omitted MRI examinations. We excluded patients for whom intervals between the examinations were disproportionately long from the beginning.

The configurations of STS on MRI were used to describe recurrences or images mimicking a recurrence. The configuration corresponds to the morphological appearance of STS. Accordingly, recurrent STS mainly presented with the following main configurations: polycyclic/multilobulated, ovoid/nodular, and streaky. Polycyclic/multilobulated STS are mainly inhomogeneous tumors, which appear multilobulated and often polycystic. Ovoid/nodular configured STS are small and round/ovoid tumors. Streaky configured STS resemble elongated scars.

### Magnetic resonance imaging

All patients were examined with a 1.5-Tesla MRI system (MAGNETOM Symphony, Siemens Healthineers, Erlangen, Germany). The MRI protocol included the following pulse sequences: T2-weighted (T2w) TSE (TE: 64–114 ms, TR: 3010–5840 ms, FOV: 22–44 cm^2^), T1-weighted (T1w) SE (TE: 10–14 ms, TR: 587–868 ms, FOV: 22–44 cm^2^), proton density-weighted (PDw) FS (TE: 26–36 ms, TR: 2740–4610 ms, FOV: 22–40 cm^2^), or Turbo-Inversion Recovery Magnitude (TIRM) (TE: 68–77 ms, TR: 4410–6980 ms, FOV: 37–45 cm^2^) and contrast-enhanced T1w SE FS (10–13 ms, TR: 533–1440 ms, FOV: 22–45 cm^2^). Slice thickness was 4–6 mm.

### Statistical data

Diagnostic accuracy was determined by calculating predictive values (positive and negative), sensitivity, specificity, and accuracy using Fisher’s exact test and 2 × 2 tables. The 95% confidence interval was determined using the Wald test. A level of *p* < 0.05 was set as statistical significance for all tests. For statistical analysis, IBM-SPSS version 26.0 software package (IBM, Armonk, NY, USA) was used.

## Results

**Fig. 2:**
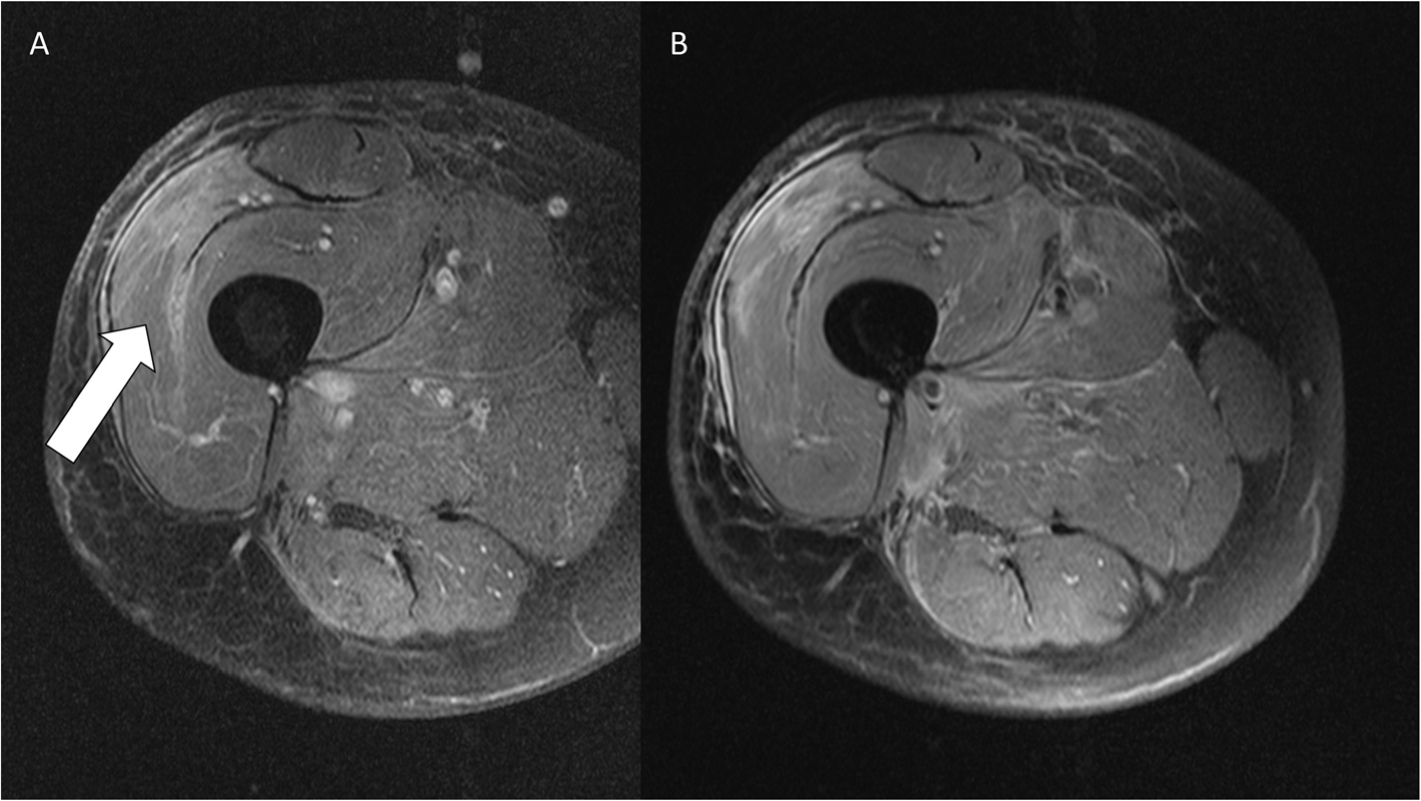
1.5-T MRI of the thigh (**a**: contrast-enhanced T1-weighted image with fat saturation in axial view; **b**: Proton density-weighted image with fat saturation in axial view). Post-treatment muscle edema (white arrow) is shown without recurrent STS

**Fig. 3:**
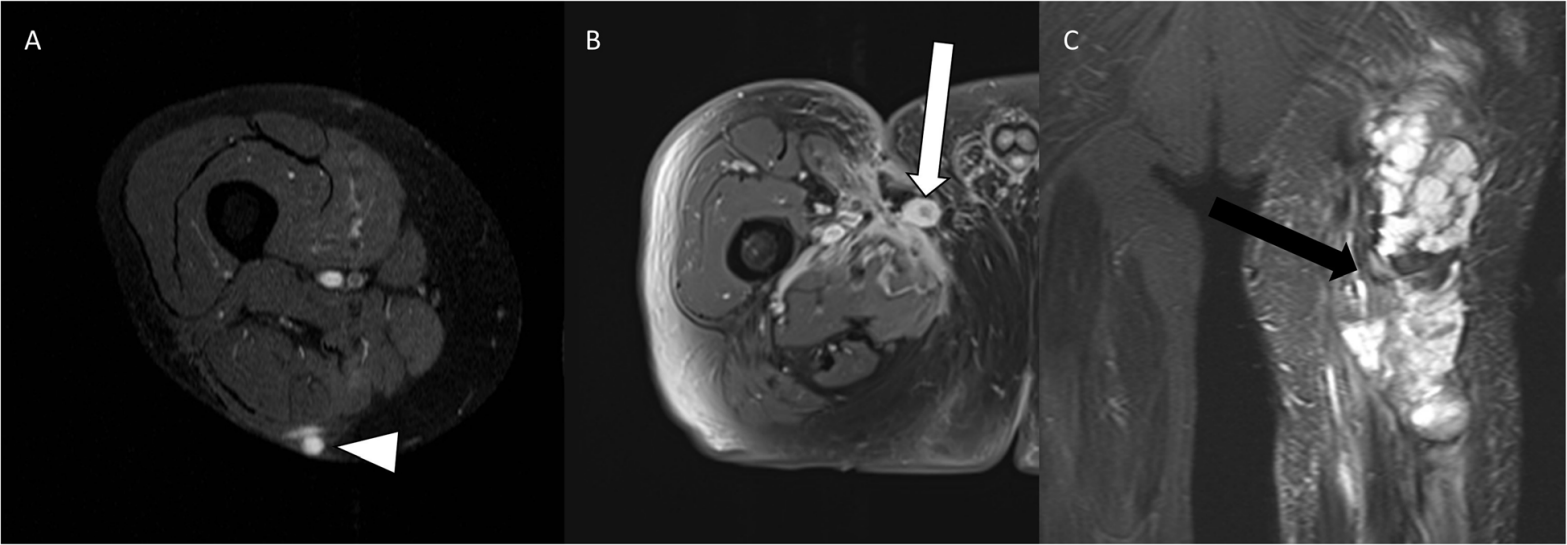
1.5-T MRI (**a**: contrast-enhanced T1-weighted image with fat suppression in axial view of the thigh; **b**: Contrast-enhanced T1-weighted image with fat suppression in axial view of the groin; **c**: Proton density weighted image with fat suppression in coronal view of the thigh). **a** shows a small nodular recurrence with slight edema (white arrowhead). **b** presents an ovoid/nodular recurrence (white arrow), which can easily be misinterpreted as a lymph node. **c** shows a multilobulated recurrence (black arrow) with adjacent edema

The median age of the patients was 55.3 years (Min.: 10, Max.: 88, SD: 18.2). Of the patients, 52.9% were male (*n* = 108; Table 1). The overall median recurrence-free follow-up interval on MRI was 39 months (Min.: 3, Max.: 161). No significant difference was observed in the median recurrence-free follow-up intervals in a comparison of patients after surgery alone (37 months) and surgery with additional radiation therapy (35 months), or in subcutaneous (37 months) and intramuscular lesions (36 months). In all, 34.8% of the patients presented with recurrences. Sixty-five follow-up MRI scans were diagnosed as true positive, 23 as false positive, 6 as false negative, and 961 as true negative (Table 2 and Fig. 2). Overall, sensitivity and specificity of MRI for detecting recurrences were 92% and 98%, respectively, with an accuracy of 97%. For intramuscular lesions the sensitivity was higher than for subcutaneous lesions (95% and 83%, respectively), at similarly high specificities (97% and 98%, respectively; Tables 3 and 4). Furthermore, the sensitivity was higher in patients after surgery alone than after surgery with additional radiation therapy (97% and 86%, respectively), at similar specificities (96% and 98%, respectively; Tables 3 and 5). The 6 false-negative results were found in streaky (*n* = 2) and small ovoid/nodular (*n* = 4) recurrences. The false-positive lesions imitated streaky (*n* = 14), ovoid/nodular (*n* = 8; Fig. 3), and polycyclic/multilobulated recurring lesions (*n* = 1). All false-positive results were found in patients in whom the primary STS was polycyclic/multilobulated in appearance. Furthermore, 22 of the 23 false-positive results were derived from patients with R0 resection (95.7%). Altogether, 92.2% of the patients underwent R0 resection.

**Table 1:** Baseline characteristics in number of patients “n”

Characteristics	n
All patients	204
Age (years)	55.3 ± 18.2
Sex	
- Female	96
- Male	108
Most common tumor sites	
- Lower extremities	101
- Upper extremities	57
- Pelvis/groin	15
- Chest wall	13
Localization	
- Subcutaneous	100
- Intramuscular	73
Histological tumor grade	
- G3	97
- G2	63
- G1	44
Margin status	
- R0	188
- R1	14
- R2	2
Treatment	
- Surgery alone	98
- Surgery plus XRT	87

**Table 2:** Summary of false-positive/−negative and true-positive/−negative results in overall MRI follow-up scans “n”

Detection of soft tissue sarcoma	Overall MRI follow-up
Total	*n* = 1055
True positive	65
False positive	23
False negative	6
True negative	961

**Table 3:** Summary of false-positive/−negative and true-positive/−negative results after resection of subcutaneous and intramuscular primary lesions, and after surgery alone and surgery with additional radiation therapy (XRT), shown in number of MRI follow-up scans “n”

Detection of soft tissue sarcoma	Subcutaneous primaries	Intramuscular primaries	Surgery alone	Surgery plus XRT
Total	*n* = 395	*n* = 371	*n* = 439	*n* = 347
True positive	15	35	35	25
False positive	12	8	15	7
False negative	3	2	1	4
True negative	365	326	388	311

**Table 4:** Calculated diagnostic accuracy (sensitivity, specificity, positive and negative predictive values, accuracy) after resection of subcutaneous and intramuscular primary lesions, with 95% confidence intervals

Diagnostic accuracy	Subcutaneous primaries	Intramuscular primaries	***p-value (sig. < 0.05)***
Sensitivity	83% (95% CI: 59–96%)	95% (95% CI: 82–99%)	*0.26*
Specificity	97% (95% CI: 95–98%)	98% (95% CI: 95–99%)	*0.43*
Positive predictive value	56% (95% CI: 41–69%)	81% (95% CI: 69–90%)	***0.04***
Negative predictive value	99% (95% CI: 98–100%)	99% (95% CI: 98–100%)	*0.48*
Accuracy	96% (95% CI: 94–98%)	97% (95% CI: 95–99%)	*0.40*

**Table 5:** Calculated diagnostic accuracy (sensitivity, specificity, positive and negative predictive values, accuracy) results after surgery alone and surgery with additional radiation therapy (XRT), with 95% confidence intervals

Diagnostic accuracy	Surgery alone	Surgery plus XRT	***p-value (sig. < 0.05)***
Sensitivity	97% (95% CI: 86–100%)	86% (95% CI: 68–96%)	*0.23*
Specificity	96% (95% CI: 94–98%)	98% (95% CI: 96–99%)	*0.36*
Positive predictive value	70% (95% CI: 59–79%)	78% (95% CI: 63–88%)	*0.27*
Negative predictive value	100% (95% CI: 98–100%)	99% (95% CI: 97–100%)	*0.41*
Accuracy	96% (95% CI: 94–98%)	97% (95% CI: 94–98%)	*0.46*

## Discussion

In our study we investigated the diagnostic accuracy of standard MRI for detecting recurring STS at a multidisciplinary sarcoma center.

STS include benign and malignant tumors and tumor-like lesions [13]. In our study we dealt with malignant lesions only. STS constitute a rare and heterogeneous group of tumors, which account for only about 1% of all malignancies [1]. The most recent histological classification of STS comes from the current WHO histological typing, in which more than 50 subtypes are described [1, 13, 14]. Previous studies on STS recurrence have reported a wide range of recurrence rates of up to 50% [15, 16]. Due to the rarity of STS, imaging studies on STS still remain scarce. Furthermore, most previous studies dealt with imaging features rather than with diagnostic accuracy. Additionally, previous diagnostic studies mostly included only small patient numbers or reviewed the available literature.

For postoperative surveillance, MRI is the imaging modality of choice and is widely used to assess STS recurrence [11, 17], as this technique has the advantage of a high soft-tissue contrast and no radiation. Nevertheless, distinguishing between post-treatment changes and recurrent STS on MRI is often reported to be challenging [18]. Previous publications have often shown that post-treatment changes and scar tissue can obscure recurring STS, which leads to unnecessary biopsies [19].

In patients with local recurrences, combined treatment with surgery and additional radiation therapy is usually chosen to improve local control [1, 20]. Nevertheless, a decision regarding the use of additional radiation therapy should be evaluated from case to case [1, 21]. Before starting the therapy, core needle biopsy is often performed to identify the pathology of the suspected lesions [22]. To start therapy quickly and to avoid unnecessary biopsy, precise postoperative MRI diagnostics are indispensable. Therefore, according to our study, it is of high clinical and diagnostic importance to determine the diagnostic reliability of MRI for postoperative surveillance of sarcoma patients in a routine clinical setting over a long time period.

In our study, both sensitivity (92%) and specificity (98%) were high overall, even after both surgery and radiation therapy and in both subcutaneous and intramuscular lesions. Indeed, sensitivity was lower after radiation therapy than after surgery alone and in subcutaneous lesions, compared to intramuscular lesions, but the sensitivity still remained at a high level. Reasons for the decreased sensitivities could lie in the increased rate of soft-tissue alterations after additional radiation therapy [19, 23] and the usually smaller sizes of the subcutaneous lesions. These two findings ultimately render it more difficult to distinguish between post-treatment changes and recurring tumor. The range of sensitivity and specificity that we found is high. However, some previous publications reported a lower specificity, ultimately leading to unneeded biopsies [11, 19, 24]. Afonso et al. described a sensitivity and specificity of only 58 and 73%, respectively, for conventional MRI [17], while for Del Grande F. et al. sensitivity and specificity of MRI were 100 and 52%, respectively, in detecting tumor recurrence in nonenhanced MRI, and 100 and 97%, respectively, in contrast-enhanced MRI [18]. In a recent review, Pennington A. et al. calculated a mean sensitivity and specificity of 88 and 86%, respectively, for local recurrences of primary vertebral tumors [25]. Other authors showed sensitivities and specificities of 64–88% and 85–96%, respectively, for MRI in detecting STS [17, 26–29]. Nevertheless, none of the previous studies investigated how MRI performed in a routine post-therapeutic clinical setting. Previous publications reported a lack of specificity of MRI in detecting recurring STS in nonenhanced T1- and T2-weighted images [19, 30, 31]. Therefore, contrast-enhanced MRI is reported to improve the diagnostic accuracy of MRI [32].

In our study, all of the patients were examined using contrast-enhanced MRI. Our data emphasize that MRI is a highly valuable imaging modality in the long-term postoperative surveillance of STS patients. Nevertheless, we found 23 false-positive and 6 false-negative results (8.5%). These 6 cases were all derived from streaky and small ovoid/nodular lesions, which were difficult to distinguish from the surrounding post-treatment tissue. A recent study showed that distinct post-therapeutic changes are the main reason for false-negative results on MRI [12]. All false-positive results were found in patients in whom the primary STS was polycyclic/multilobulated. This may well be due to the fact that polycyclic/multilobulated primary tumors are larger than other STS configurations in the mean and therefore perhaps lead to more heterogeneous post-treatment tissue changes. Furthermore, 22 of the 23 false-positive results were derived from patients with R0 resection. This finding is in contrast to a recent study showing that microscopic positive margins were the main reason for false-positive results [12]. Therefore, radiologists should pay particular attention to patients in whom the primary STS was polycyclic/multilobulated in shape and should carefully screen the soft tissue for streaky or small ovoid/nodular recurrences. However, this fact also demonstrates the limitations of MRI surveillance. In cases of recurrent lesions with a small and not clearly delimited appearance, it may become difficult to correctly detect a recurring tumor in the surrounding tissue. Therefore, for suspected recurrence or unclear cases, the subsequent MRI follow-up examinations should take place after 3 to 6 months. The justification for this time is also evident from our study as the two readers did find all false-negative recurrences retrospectively by reviewing the subsequent MRI follow-ups.

Our study has some limitations, as it is a single-center study with a retrospective design. Nevertheless, we could include 204 patients in a 12-year survey with a total of 1055 MRI follow-up scans. Another limitation is the verification of the true-positive/−negative and false-positive/−negative results. Biopsy was only performed in patients in whom recurrence was suspected. In the other cases, radiologists evaluated whether recurrences were overlooked or not. Therefore, we cannot completely rule out that individual findings might be false.

## Conclusion

MRI shows a high diagnostic accuracy for detecting recurring STS in the long term, with a high sensitivity (92%) and specificity (98%). After resection of subcutaneous primary tumors and after radiation therapy, the sensitivity decreases to 83 and 86%, respectively, compared to intramuscular lesions and surgery alone (95 and 97%, respectively). Radiologists should pay particular attention to patients in whom the primary tumor was polycyclic/multilobulated in appearance and should carefully screen the post-treatment soft tissue for streaky and small ovoid/nodular recurrences, which are often difficult to distinguish from post-treatment changes.

## Data Availability

The datasets used and/or analyzed during the current study are available from the corresponding author on reasonable request.

## Abbreviations

MRI: Magnetic resonance imaging
STS: Soft-tissue sarcoma
